# Water-Dispersed Poly(p-Phenylene Terephthamide) Boosting Nano-Al_2_O_3_-Coated Polyethylene Separator with Enhanced Thermal Stability and Ion Diffusion for Lithium-Ion Batteries

**DOI:** 10.3390/polym11081362

**Published:** 2019-08-18

**Authors:** Haopeng Cai, Guoping Yang, Zihan Meng, Xue Yin, Haining Zhang, Haolin Tang

**Affiliations:** 1 School of Materials Science and Engineering, Wuhan University of Technology, Wuhan 430070, China; 2 State Key Laboratory of Advanced Technology for Materials Synthesis and Processing, Wuhan University of Technology, Wuhan 430070, China

**Keywords:** Lithium-ion batteries, Poly(p-phenylene terephthamide), Nano-Al_2_O_3_, Thermal stability, Ion diffusion

## Abstract

Polyethylene (PE) membranes coated with nano-Al_2_O_3_ have been improved with water-dispersed poly(p-phenylene terephthamide) (PPTA). From the scanning electron microscope (SEM) images, it can be seen that a layer with a honeycombed porous structure is formed on the membrane. The thus-formed composite separator imbibed with the electrolyte solution has an ionic conductivity of 0.474 mS/cm with an electrolyte uptake of 335%. At 175 °C, the assembled battery from the synthesized composite separator explodes at 3200 s, which is five times longer than the battery assembled from an Al_2_O_3_-coated polyethylene (PE) membrane. The open circuit voltage of the assembled battery using a composite separator drops to zero at 600 s at an operating temperature of 185 °C, while the explosion of the battery with Al_2_O_3_-coated PE occurs at 250 s. More importantly, the interface resistance of the cell assembled from the composite separator decreases to 65 Ω. Hence, as the discharge rate increases from 0.2 to 1.0 C, the discharge capacity of the battery using composite separator retains 93.5%. Under 0.5 C, the discharge capacity retention remains 99.4% of its initial discharge capacity after 50 charge–discharge cycles. The results described here demonstrate that Al_2_O_3_/PPTA-coated polyethylene membranes have superior thermal stability and ion diffusion.

## 1. Introduction

Despite the fact that lithium-ion batteries (LIBs) have been widely used in many aspects of our lives, from portable electronic devices to electric vehicles, safety during the operation of LIBs still remains a great challenge [1,2,3,4,5]. The safety issues of LIBs are considerably associated with the thermal stability of electrodes, electrolytes, and particularly separators [6,7]. In a battery system, the membrane separator plays a significant role in preventing electronic contact between the anode and cathode, concurrently acting as an electrolyte reservoir to enable ion transport between the anode and the cathode [8,9,10,11,12].

Nano aluminum oxide (nano-Al_2_O_3_) has been realized as a promising ceramic coating due to its high thermal stability and chemical inertness [13,14,15]. In the scientific community, there are numerous researchers using it to solve the related battery safety problems. For example, curable copolyester and Al_2_O_3_ were coated on the surface of a pristine polyethylene (PE) separator. When exposed to 170 °C, the shrinkage of the composite-coated membrane was about 13% [16]. Wu et al. fabricated a high-safety Al_2_O_3_-based composite separator via tip-induced electrospinning and dip-coating. Kept at a high temperature of 140 °C for an hour, the as-synthesized separators mechanically contracted less than 2% [17]. Shen et al. developed a core-shell nanofiber separator by atomic layer deposition of 30 nm Al_2_O_3_ on the electrospinning nonwoven fiber. The membranes showed a slight shrinkage in temperatures up to 200 °C. Moreover, the composite separator has a high electrolyte uptake and ionic conductivity [18]. Considering the cost of commercialization, the composite separator produced by electrospinning cannot be used on a large scale.

In the face of global warming, climate anomalies, and other natural disasters, people realize the importance of environmental protection. In 2018, the world consumption of LIB membranes was about 8.768 billion square meters, which caused terrible damage to the environment. Based on methacrylate and cellulose, Chiappone et al. prepared a membrane for LIBs by photo-polymerization and “in situ” grafting [19]. The prepared membrane is not only ecofriendly, but also has eximious mechanical and electrochemical properties. As we know, there are now as many as 30,000 kinds of organic solvents, and their consumption is too large to be estimated. Moreover, organic solvents not only damage the environment but also harm the health of scientific researchers and workers. Influenced by the idea of environmental protection, we developed a coating slurry with water dispersion. Furthermore, Poly(p-phenylene terephthamide)s (PPTAs) are five to six times stronger than steel wire, and their thermal decomposition temperature is about 600 °C. PPTA is a superior fibrous material that can be used to improve the separator in LIBs. Herein, a mixture of a water-dispersed PPTA and nano-Al_2_O_3_ was applied as a coating material to improve the thermal stability and ion diffusion capability of PE membrane separators. From the SEM image, it can be seen that a layer with a honeycombed porous structure was formed on the membrane. The honeycombed porous structure resulted in an increased electrolyte uptake. Accordingly, the improved ionic conductivity and the time of battery explosion was substantially extended. Moreover, the thus-assembled battery exhibited a much higher discharge capacity (particularly at high current density) and the most robust cycling performance compared with batteries assembled using a pristine PE membrane separator and the often-used composite PE separator coated with nano-Al_2_O_3_. After 50 cycles, the capacity retention of the PE coated with water dispersed Al_2_O_3_/PPTA was 99.4% under a 0.5 C discharge rate. The pristine PE separator’s retention was 92.8% under the same conditions. Hence, the PE-based composite membrane separator coated with Al_2_O_3_/PPTA can practically address the existing safety and ion diffusion issues of LIBs. 

## 2. Experimental

### 2.1. Preparation of Composite Separators

To prepare casting dispersion, 1 g of aramid fibers (X-FIPER New Material Co., Ltd., Shanghai, China) was dissolved in 60 mL dimethyl sulfoxide (DMSO). After the addition of 6 mL of deionized water and 3 g KOH, the mixture was stirred at 50 °C for 96 h. Then, 2 mL propargyl bromide was added in the above mixture and heated at 30 °C for 16 h. After water was added to the reaction system, precipitates of the aramid nanofibers with active functional groups were acquired. The obtained functional aramid nanofibers were dissolved in a mixed system of DMSO/KOH/H_2_SO_4_. Then, these nanofibers were added under a deoxygenized condition to obtain a hydrophilic PPTA. Solid particles of hydrophilic PPTA were collected via water dialysis. Nano-Al_2_O_3_ (Shanghai Macklin Biochemical Co., Ltd., Shanghai, China) and the prepared hydrophilic PPTA solid particles (2:1, wt. ratio) were dispersed in deionized water and stirred at room temperature for 48 h to obtain the casting dispersion. The composite membrane separator was finally fabricated through casting the dispersion on 9 μm thick PE membrane separator (CubeEnergy, Dongguan, China) using a coating machine (CubeEnergy). The thickness of the coating layer was about 3 μm.

### 2.2. Characterization of the Composite Separators

To demonstrate the presence of poly(p-phenylene terephthamide) on the separator, Fourier transform infrared (FTIR) spectra were recorded on a Nicolet Nexus 470 spectrometer in the range of 400–4000 cm^−1^ with a resolution of 4 cm^−1^. The morphology and microstructures of membrane separators were investigated using a field emission scanning electron microscope (FE-SEM, QUANTA, FEI) with an operating voltage of 5.0 kV. The corresponding chemical composition was characterized by energy dispersive X-ray spectroscopy (EDX, Oxford Instruments X-ray Microanalysis 1350, Shanghai, China). The electrolyte uptake was calculated by the weight difference before and after soaking the membrane separators into liquid electrolyte (1.0 M lithium hexafluorophosphate (LiPF_6_) in a mixed solvent of ethylene carbonate (EC), diethyl carbonate (DEC), and dimethyl carbonate DMC with a volume ratio of 1:1:1) separately for 30 min, followed by wiping the liquid on the surface with filter paper.

Thermogravimetric analysis (NETZSCH STA 449F3 STA449F3A-1383-M, Bavaria, Germany) was performed under air flow from 40 to 800 °C, with a heating rate of 10 °C per minute. Thermomechanical (TMA) tests were performed on TMA analyze (TMA202, NETZSCH, Bavaria, Germany) at a ramping rate of 5 °C/min under a nitrogen purge from 30 to 250 °C. The thermal shrinkage of separators was tested by placing the installed relevant membranes on two glasses in an oven at various temperatures from 125 to 185 °C for 30 min. 

In order to test the cell at high temperature, the cell was assembled by sandwiching the separator between a MCMB (Mesophase Carbon Micro Beads) anode and a LiFePO_4_ (Kejing Zhida Co., Ltd., Shenzhen, China) cathode, followed by activation by filling the liquid electrolyte (1.0 M LiPF_6_ in mixed solvent of EC, DEC, and DMC with volume ratio of 1:1:1), which was sealed in a CR2016 shell with the pressure of 50 kg/cm^2^ by using a sealing machine (MSK-110, MTI Corp., Shenzhen, China).The batteries were first charged to a 75% state of charge using cell test equipment (CT2001A, LAND Electronics, Wuhan, China). The cells were left in open circuit for 24 h and then were heated in an oven to test the open circuit voltage (OCV) at high temperatures using an electrochemical workstation (CHI604D, CH Instruments, Shanghai, China).

The ionic conductivity of the membrane separators filled with electrolyte solutions of 1.0 M LiPF_6_ in a mixed solvent of EC, DEC, and DMC with a volume ratio of 1:1:1 was conducted on an impedance analyzer (CHI604D, CH Instruments) using circular membranes with diameters of 17.0 mm. The membranes were sandwiched by two stainless steel electrodes. The tested frequency ranges from 1.0 to 10^6^ Hz with a signal amplitude of 5 mV. Ionic conductivity (σ) was calculated from the obtained resistance by σ = d/(R × S), where d is the thickness of the membranes, R is the bulk resistance, and S is the area of the electrode. The activation energy was acquired by measuring the ionic conductivities of the membrane at different temperatures from 25 to 100 °C. The activation energy was calculated using the Arrhenius, σ = A exp (−E_a_/RT), where σ is the ionic conductivity, A is the pre-exponential factor, R is the gas constant, and T is the temperature (K). The electrochemical stability of the membranes soaked with the electrolyte was determined by liner sweep voltammetry (LSV) from 2.5 to 7.5 V, with a voltage scan rate of 2 mVs^−1^. 

The battery performance was investigated using battery test equipment (CT2001A, LAND Electronics). The half-cell was assembled by sandwiching the separator between a lithium anode and a LiFePO_4_ (Kejing Zhida Co., Ltd) cathode and then activated by filling it with a liquid electrolyte (1.0 M LiPF_6_ in mixed solvent of EC, DEC, and DMC with volume ratio of 1:1:1), followed by being sealed in a CR2016 shell with a pressure of 50 kg/cm^2^ by using a sealing machine (MSK-110, MTI Corp.). The discharge current densities varied from 0.2 to 1 C under a voltage range between 2.5 V and 4 V. The cells were cycled at a fixed charge/discharge current density of 0.5 C. The interfacial resistance of the different membranes sandwiched between the lithium metal electrode was measured by electrochemical impedance spectroscopy (EIS) at an amplitude of 5 mV over a frequency range of 0.01 to 10^5^ HZ.

## 3. Results and Discussion

**Figure 1:**
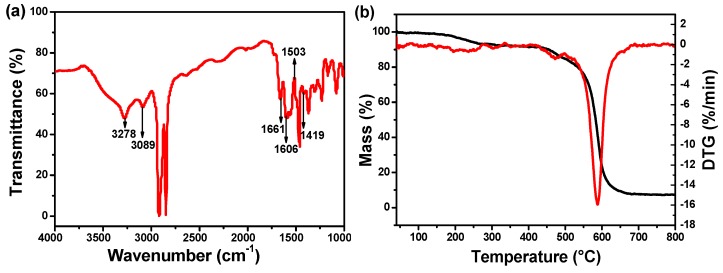
(**a**) Fourier transform infrared (FTIR) spectrum of Al_2_O_3_/poly(p-phenylene terephthamide) (PPTA)-coated polyethylene (PE) separator. (**b**) TG-DTG of solute of functional PPTA.

The FTIR spectrum was recorded to investigate the chemical composition of the formed composite membrane separator, as shown in Figure 1a. The appearance of characteristic absorption bands of –CO–NH– at 3278 cm^−1^, 1661 cm^−1^ and 1419 cm^−1^, confirmed the existence of PPTA. The absorption bands attributed to benzene skeleton vibration appeared at 1606 cm^−1^, 1503 cm^−1^, and the absorption band at 3089 cm^−1^ is assigned to the C–H stretching vibration on the benzene ring. Thermal stability of functionalized PPTA was investigated by thermalgravimetric analysis (Figure 1b). It is apparent that the decomposition of functionalized PPTA starts at 580 °C.

**Figure 2:**
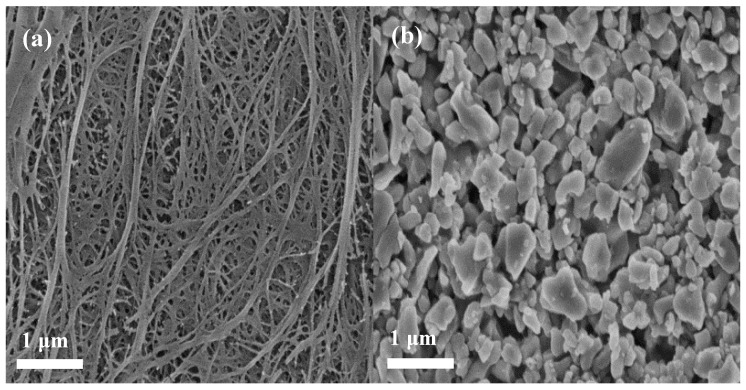

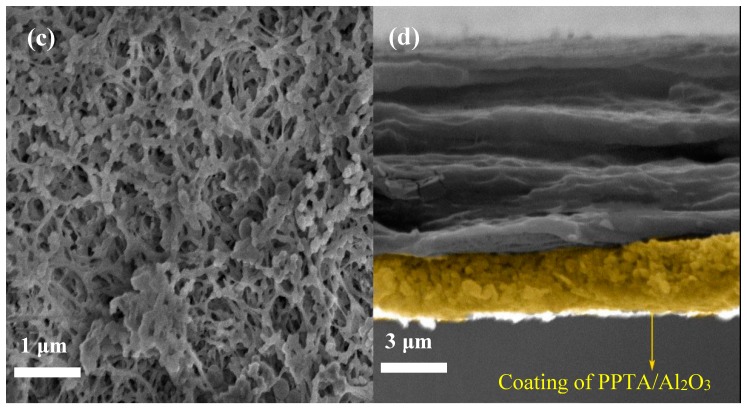
Field emission scanning electron microscope (FE-SEM) images (surface) for the PE separator (**a**), Al_2_O_3_-coated PE, (**b**) and Al_2_O_3_/PPTA-coated PE (**c**), respectively. Cross-section photograph (**d**) of the Al_2_O_3_/PPTA-coated PE membrane.

**Figure 3:**
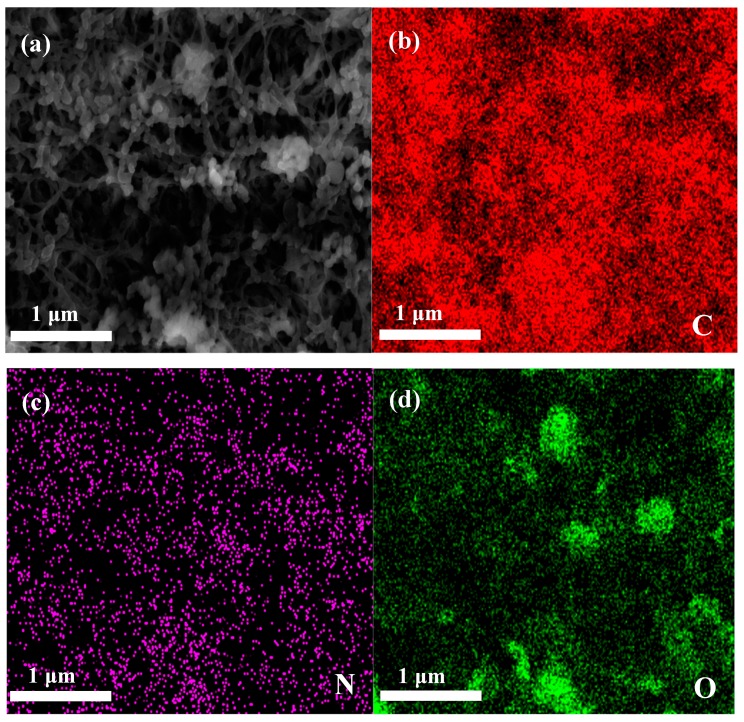
(**a**) Represents the region of the elemental map. (**b**–**d**) Elemental mapping of C, N, and O.

The surface morphology and microstructure of the applied membrane separators were investigated by FE-SEM, as shown in Figure 2. It can be seen that the pristine PE membrane separator (Figure 2a) exhibits a clear porous structure whereas the relatively loose uniform coating layers were observed for composite membrane separators (Figure 2b,c). Careful observation of the SEM images revealed that nano-Al_2_O_3_ forms porous structures with aramid fibers. Of course, porous structures are beneficial to the electrolyte uptake of the membrane, and a smaller gap can enhance the diffusion efficiency of lithium ions and adsorbed anions. An image of a cross-section (Figure 2d) indicates that the Al_2_O_3_/PPTA coating adheres to the membrane and thickness of the coating around 3 μm. The elemental proportion of the Al_2_O_3_/PPTA coating is listed in Table 1. Elemental mappings from SEM images (Figure 3) demonstrate that PPTA and Al_2_O_3_ are evenly distributed on the membrane separator.

**Figure 4:**
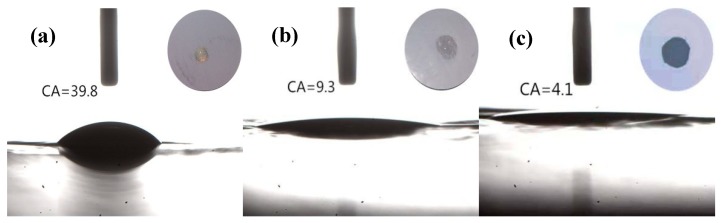
Static contact angles of the pristine PE separator (**a**), Al_2_O_3_-coated PE, (**b**) and Al_2_O_3_/PPTA-coated PE (**c**).

The quick and high-wettability of separators toward characteristic cell electrolytes is of great importance for the electrochemical performance and assembling process of batteries [20,21,22,23,24]. To quantitatively assess the electrolyte wettability of the membranes, the static contact angles were measured and the according photographs are shown in Figure 4. Visual inspection already suggested that the electrolyte solution can easily wet the Al_2_O_3_/PPTA-coated PE membrane. More specifically, the contact angle of Al_2_O_3_/PPTA-coated PE membrane in contact with the electrolyte solution is about 4.1°, whereas they are about 39.8° and 9.3° for the pristine PE membrane and the Al_2_O_3_-coated PE membrane, respectively. Such a decrease in electrolyte contact angle for the Al_2_O_3_-coated PE and Al_2_O_3_/PPTA-coated PE is attributed to nano-Al_2_O_3_ particles with an outstanding ability to absorb electrolytes. Particularly, the finer voids of the Al_2_O_3_/PPTA coating also contributed to the absorption of the electrolyte. The results suggest that modification of the PE membrane with water-dispersed PPTA is an effective strategy to enhance the wetting property of the Al_2_O_3_-coated PE separator.

**Figure 5:**
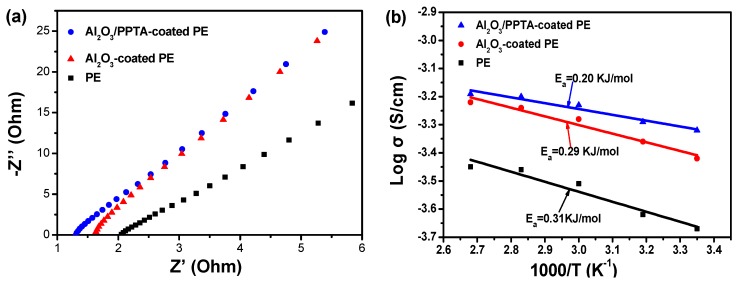
The electrochemical impedance spectra (**a**) of different separators soaked with electrolytes at 25 °C. The temperature dependence of ionic conductivity (**b**) of different separators soaked with electrolyte.

Generally, the membrane with a low electrolyte contact angle implies high electrolyte uptake and accordingly high ionic conductivity [25,26]. The calculated electrolyte uptake (Table 2) was about 335% for Al_2_O_3_/PPTA-coated PE membrane, much higher than that of the pristine PE membrane (91%) and nano Al_2_O_3_-coated PE membrane (186%). The ionic conductivity of the according separator filled with the electrolyte is an indicator of whether the membrane can be applied for practical LIB applications. The ionic conductivity of membrane separators and electrochemical impedance values (Table 2) revealed that the Al_2_O_3_/PPTA-coated membrane exhibited the highest ionic conductivity (0.474 mS/cm) compared with the pristine PE membrane (0.216 mS/cm) and Al_2_O_3_-coated PE membrane (0.376 mS/cm), due to the electrolyte affinity of the nano Al_2_O_3_ and its special structure that is conducive to the diffusion of lithium ions. It is of great significance to explore the ionic conductivity of the membrane at different temperatures for its application. The ionic conductivity of the membranes soaked with electrolytes generally increased with an increase in temperature (Figure 5b). The ionic conductivity of the Al_2_O_3_/PPTA-coated PE membrane soaked with electrolytes increased from 0.474 mS/cm at 25 °C to 0.647 mS/cm at 100 °C. Comparing the slope of the curves of the controlling samples, the Al_2_O_3_/PPTA-coated PE membrane exhibited the lowest activation energy. This phenomenon may derive from the glass transition of PPTA at a higher temperature, which generated more voids conducive to the passage of lithium ions.

**Figure 6:**
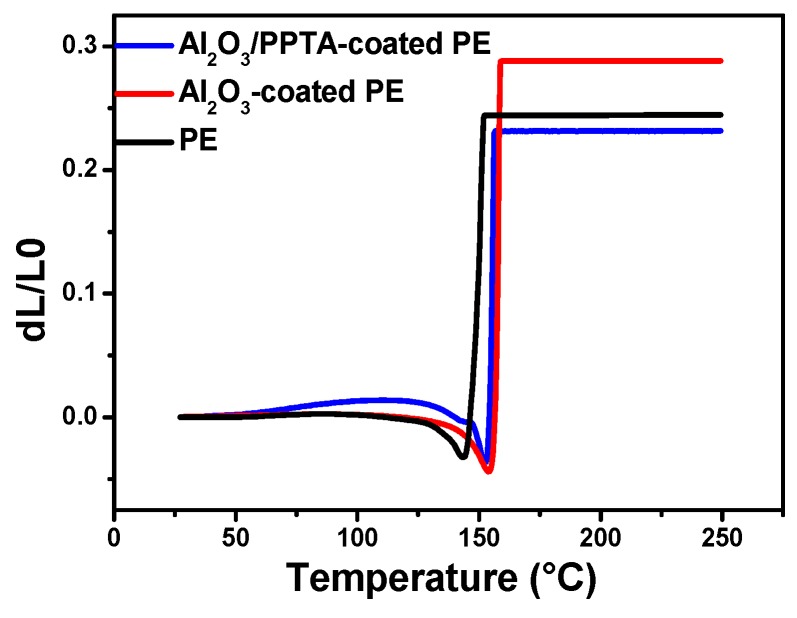
Thermomechanical behavior of the pristine PE, Al_2_O_3_-coated PE, and Al_2_O_3_/PPTA-coated PE membrane.

Figure 6 shows the dimensional changes of three different membranes as a function of temperature. It is clearly observed that the TMA curve of the Al_2_O_3_/PPTA-coated PE membrane has a slightly lower degree of thermal shrinkage. Compared to the others separator, the maximum length reduction of the composite separator was far lower.

**Figure 7:**
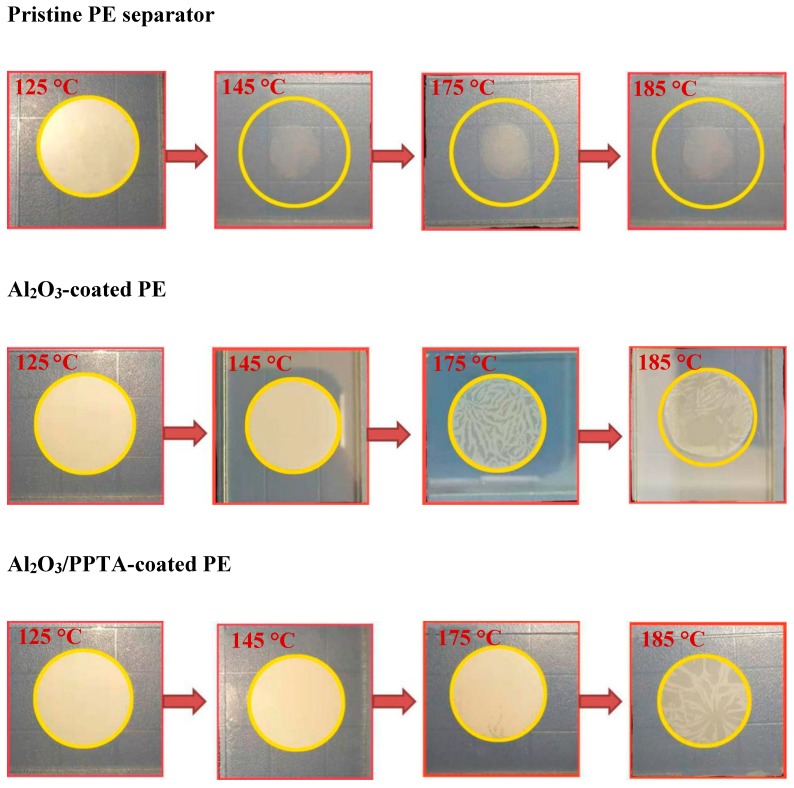
Photographs of shrinkage and color changes of the pristine PE separator, Al_2_O_3_-coated PE, and Al_2_O_3_/PPTA-coated PE at different temperatures.

The thermal dimensional stability for all three tested membrane separators is quite different, as illustrated in Figure 7. The remarkable thermal stability of the separator will largely eliminate the safety concerns of lithium ion batteries when working at high temperatures. The dimensional stability of membrane separators was measured by placing the sandwiched membrane separators between two glass slides in an oven at various temperatures from 125 to 185 °C for 0.5 h. It can be clearly seen that the pristine PE and Al_2_O_3_-coated PE membranes started to shrink at 145 and 185 °C, respectively. Both membranes became transparent at 185 °C. However, for the Al_2_O_3_/PPTA-coated membrane, no obvious shrinkage was observed at temperatures below 175 °C, and the color of the membrane only slightly changed at 185 °C. Hence, it can be concluded that the thermal dimensional stability of the Al_2_O_3_-coated PE membrane separators can be significantly improved after being coated with water-dispersed poly(p-phenylene terephthamide). This superior ability to maintain the integrity of the separator derives from the properties of the nano-sized Al_2_O_3_ and the unique structure of the tightly combining PPTA fiber with the nanoparticles.

**Figure 8:**
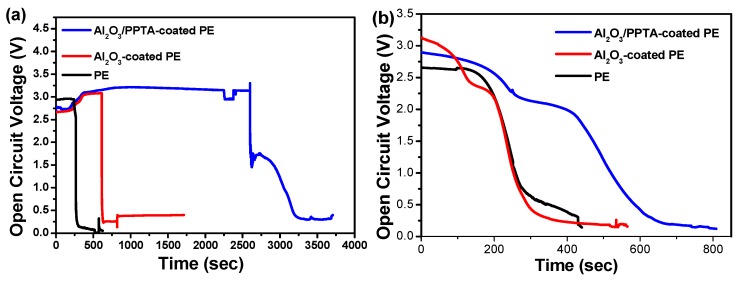
Variation of OCV values as a function of operating time for assembled LiFePO_4_/separator/MCMB batteries using PE, Al_2_O_3_-coated PE, and Al_2_O_3_/PPTA-coated PE membranes soaked with electrolytes at different temperatures: 175 °C (**a**) and 185 °C (**b**).

It has been reported that a serious shrinkage of separators can lead to direct contact between the cathode and the anode, generating a sudden decrease in OCV [27,28,29]. The stability of the OCV values of the assembled battery with different membrane separators was investigated at different temperatures, as displayed in Figure 8. With an increase in operating temperature to 175 °C, the explosion of the battery assembled from the Al_2_O_3_/PPTA-coated PE membrane occurred after 3200 s (Figure 8a), much longer than that of the battery assembled from the pristine PE separator (250 s) and the battery assembled from the Al_2_O_3_-coated PE membrane separator (730 s). With a further increase in operating temperature to 185 °C (Figure 8b), the OCV of the battery assembled from the Al_2_O_3_/PPTA-coated PE membrane separator dropped to zero at around 600 s, much longer than that of the batteries assembled from pristine PE and Al_2_O_3_-coated PE separators. Considering the safety of LIBs, the short circuit issues are mainly induced by the poor thermal stability of the membrane. The designed superior heat-resistant separator of PE coated with water-dispersed Al_2_O_3_/PPTA is practically applicable for various special applications, such as aerospace crafts and nuclear power factories.

**Figure 9:**
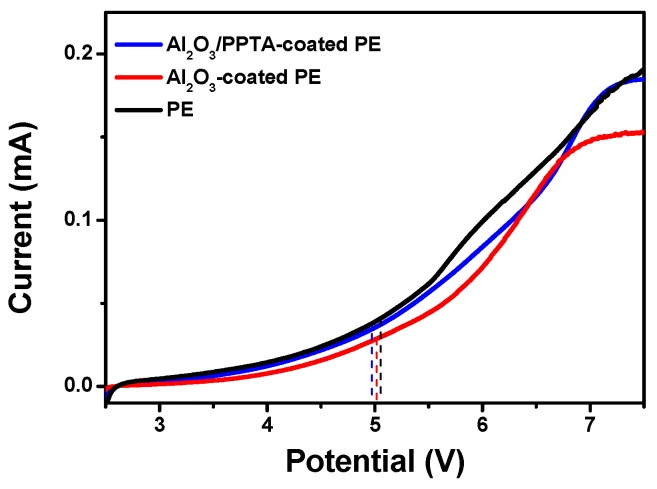
LSV of different separators.

Figure 9 shows the electrochemical stability window of the applied membrane separators evaluated by LSV [30,31]. A very low background was scaled below 5.0 V, followed by a little increase in current flow, which demonstrated the onset potential of the electrochemical decomposition of the electrolyte. This phenomenon indicated that those investigated separators have sufficient electrochemical stability to be charged and discharged against the anodic potential applied in this work (4.5 V).

**Figure 10:**
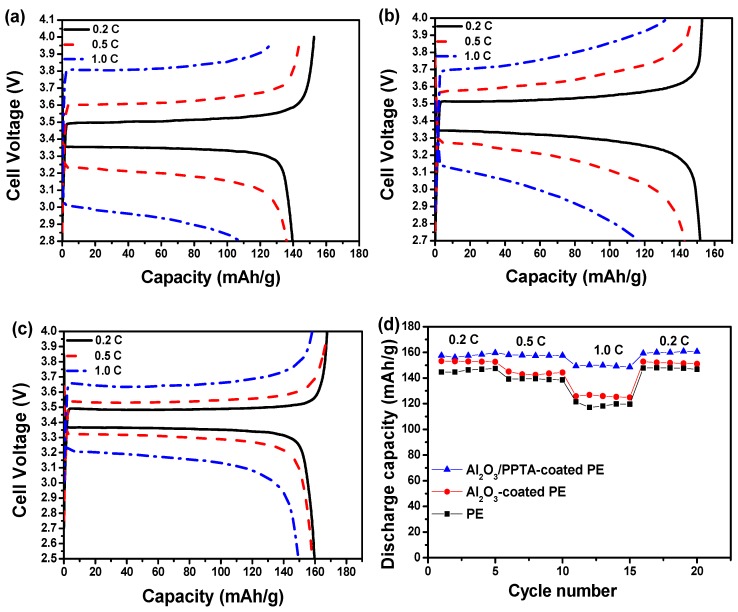

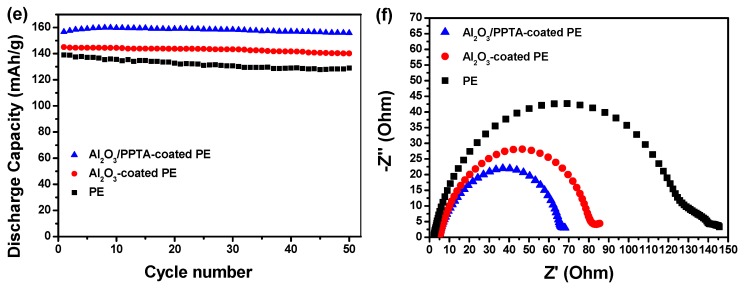
The charge–discharge profiles of lithium ion batteries assembled from pristine PE (**a**), Al_2_O_3_-coated PE (**b**), and Al_2_O_3_/PPTA-coated PE (**c**) separators. (**d**) The rate capability of the batteries assembled from different membrane separators, as indicated in Figure 9. (**e**) Cycle performance of the batteries assembled from different membrane separators at 0.5 C. (**f**) Nyquist plots for the cells with pristine PE, Al_2_O_3_-coated PE, and Al_2_O_3_/PPTA-coated PE, respectively.

The electrochemical performance of the thus-assembled LIBs using different membrane separators was finally investigated at 25 °C using a lithium metal plate as an anode and LiFePO_4_ as a cathode. From the initial charge–discharge profiles of the assembled batteries, it can be seen that the initial discharge capacities at 0.2 C for the batteries assembled from pristine PE (Figure 10a), Al_2_O_3_-coated PE (Figure 10b), and Al_2_O_3_/PPTA-coated PE (Figure 10c) membrane separators are 144.6, 153.1, and 157.6 mAhg^−1^, respectively. The relatively low initial discharge capacity for the battery assembled from the Al_2_O_3_-coated PE membrane separators indicates that Al_2_O_3_ and PE are less compatible, leading to an increased interfacial resistance (Figure 10b) and, accordingly, a decreased discharge capacity. Such a difference in the initial discharge capacity could be possibly explained by the different charge–transfer resistance (R_ct_). As shown in Figure 10f, the order of the t R_ct_ values is: Al_2_O_3_/PPTA-coated PE (65 Ω) < Al_2_O_3_-coated PE (80 Ω) < PE (130 Ω). The lowest R_ct_ of the battery based on the Al_2_O_3_/PPTA-coated PE could be ascribed to the higher electrolyte uptake and the unique microstructure conducive to the diffusion of lithium ions. With the increase in operating rate, the discharge capacities for the assembled batteries from the different membrane separators are quite different, as shown in Figure 10d. It is evident that, with the increase in discharge rate from 0.2 to 1.0 C, the discharge capacity of the battery assembled from the Al_2_O_3_/PPTA-coated PE membrane separator retains about 93.5% of the capacity at 0.2 C, whereas the retention values are only about 80.9% and 82.8% for batteries assembled from pristine PE and Al_2_O_3_-coated PE membrane separators, respectively. Figure 10e illustrates the cycling performance of batteries assembled from three different separators at 0.5 C. For the battery assembled from the pristine PE membrane separator, the apparent discharge capacity loss can be observed, and the discharge capacity decreased to 129.0 mAhg^−1^ after 50 cycles with a capacity retention of 92.8%. For batteries assembled from Al_2_O_3_/PPTA-coated PE and Al_2_O_3_-coated PE membrane separators, the discharge capacity reduced to 155.9 mAhg^−1^ and 140.1 mAhg^−1,^ with the capacity retention of 99.4% and 96.6%, respectively. The improvement in the cycling performance of the battery assembled from the Al_2_O_3_/PPTA-coated PE membrane separator can probably be attributed to the increased electrolyte uptake, sharply reduced interfacial resistance, and excellent ionic conductivity induced by its great liquid electrolyte affinity. More importantly, the microstructure with a fine uniform distribution of voids enhanced the lithium ion diffusion capacity and tortuosity of the membrane micropores and reduced the mechanical micro-short circuit in the circulation. Thus, it can be concluded that the Al_2_O_3_/PPTA-coated PE membrane is a promising membrane separator for LIBs with improved electrochemical performance [32,33,34,35].

## 4. Conclusions

New composite membrane separators are fabricated by coating the mixture of water-dispersed poly(p-phenylene terephthamide) with nano-Al_2_O_3_ on the surface of polyethylene membranes. The improved electrolyte affinity with a membrane separator by functional polar groups on PPTA can facilitate lithium ion transport even under a high current density, leading to reduced interfacial resistance and improved ionic conductivity. The as-prepared composite membrane separator exhibited an improved thermal shrinkage stability compared with the pristine PE separator and the often-used Al_2_O_3_-coated PE separator. The thus-assembled battery was much safer than the battery assembled from the Al_2_O_3_-coated PE separator, as evidenced by the longer time for the open circuit voltage drop and the explosion that occurred at temperatures beyond 175 °C. Furthermore, the assembled battery from the composite membrane separator exhibited superior cycle stability due to the facilitated lithium ion transport across the separator, retaining 99.4% of its initial discharge capacity after 50 charge–discharge cycles. Hence, Al_2_O_3_/PPTA-coated PE membrane separators can be applied in efficient lithium ion batteries with high safety.

## Figures and Tables

**Table 1:** Energy dispersive X-ray (EDX) analysis of the Al_2_O_3_/PPTA-coated PE separator.

Element	Wt%	At%
C	70.75	78.24
N	1.81	1.72
O	19.30	16.03
Al	8.14	4.01

**Table 2:** The electrochemical impedance, ionic conductivity, and electrolyte uptake of different membranes soaked with electrolytes at 25 °C.

	Resistance, Ω	Ionic Conductivity, mS/cm	Electrolyte Uptake, %
PE	2.02	0.216	91
Al_2_O_3_-coated PE	1.55	0.376	186
Al_2_O_3_/PPTA-coated PE	1.23	0.474	335
